# In Vitro Assessment of the Effectiveness of Mineral Adsorbents in Sequestering Boar Taint Compounds

**DOI:** 10.3390/ani15060765

**Published:** 2025-03-07

**Authors:** Sanghyuk Park, James Squires

**Affiliations:** Department of Animal Biosciences, University of Guelph, Guelph, ON N1G 2W1, Canada; peter.park@skretting.com

**Keywords:** boar taint, androstenone, skatole, activated charcoal, bentonite, diatomaceous earth, spent filter aid, hydrated sodium–calcium aluminosilicate

## Abstract

The utility of four mineral adsorbents as potential feed additives to bind the boar taint compounds, androstenone and skatole, was assessed with an in vitro system. The adsorbents were bentonite (BNT), diatomaceous earth (DE), spent filter aid (SFA) and hydrated sodium–calcium aluminosilicate (HSCAS), with activated charcoal (AC) as a positive control. The binding capacity (B_max_) and binding affinity (K) of androstenone (AND), estrone (E1), estrone sulfate (E1S), and skatole were estimated using the modified Michaelis–Menten kinetics. The Langmuir and Freundlich isotherm models were also used to assess the adsorption behaviour. The B_max_ values with AND were 77.7 ± 1.12%, 71.9 ± 1.93%, 55.0 ± 7.85%, and 69.5 ± 1.39% for BNT, DE, SFA, and HSCAS, respectively, with no differences in the binding affinity K (*p* > 0.05). All the mineral adsorbents had very low binding with E1S. SFA bound skatole with a B_max_ of 89.9 ± 1.09%, while the B_max_ values for skatole binding by BNT, DE and HCAS were approximately 15%. Most adsorbent–adsorbate complexes fit best with the Freundlich isotherm model. We conclude that all four mineral adsorbents bound androstenone, but not E1S, and only SFA effectively bound skatole. This suggests that SFA may act as a selective dietary binding agent to control boar taint, but further research using animal models is needed to explore the utility and selectivity of these adsorbents as feed additives to control boar taint.

## Simple Summary

Male piglets are routinely castrated to prevent boar taint, an off-odour and off-flavour in the meat from male pigs that is caused by high levels of two compounds, androstenone and skatole. However, castration prompts serious animal welfare concerns and reduces the efficiency and sustainability of pork production systems, so alternatives to castration for the control of boar taint are needed. Proof of concept studies using activated charcoal as a dietary binding agent showed that it is able to reduce boar taint, but more cost-effective and selective binding agents for boar taint compounds with potential to be approved for use in animal feeds are needed. Here, we investigated the mineral-based adsorbents bentonite, diatomaceous earth, spent filter aid and hydrated sodium–calcium aluminosilicate as alternatives to activated charcoal as potential binding agents to reduce boar taint in male pigs. We found that all four mineral adsorbents selectively bound androstenone, but only spent filter aid effectively bound skatole. This suggests that spent filter aid may act as a selective dietary binding agent to control boar taint. Provided that animal performance is not compromised, as assessed by in vivo feeding trials, this dietary approach for controlling boar taint is a promising and appealing alternative to castration for both consumers and producers.

## 1. Introduction

A major obstacle when raising intact male pigs (boars) for the North American, European, Asian and other global markets is the potential for boar taint, which is an undesirable odour and taste in pork from some intact males. Boar taint is caused primarily by the accumulation of two compounds in adipose tissue: 5α-androst-16-ene-3-one (androstenone), which is a 16-androstene steroid sex pheromone produced by the testis, and 3-methylindole (skatole), which is produced from the degradation of tryptophan by the gut microbiota. In most countries, surgical castration is a routine practice for mitigating this issue. However, castration is a major animal welfare concern [1], and removing the source of endogenous anabolic steroids also decreases the superior growth performance of intact males, thereby justifying the development of alternative strategies to prevent boar taint [2,3].

Androstenone, along with androgens and estrogens, is metabolized in the liver to form sulfo-conjugates and glucuronyl-conjugates [4,5]. Steroid conjugates then transit to the digestive tract via the bile and, following deconjugation by the gut microflora, are reabsorbed back into the body via enterohepatic circulation [6]. Based on this theory for androstenone, one proposal to decrease its levels may be including adsorbent materials to finishing pig diets, potentially binding to androstenone in the gut and preventing its reuptake. In a pilot study by our group, activated charcoal (AC) added to the feed effectively decreased the fat androstenone concentrations [7], but further detailed studies are needed to determine the mechanism and assess the efficacy of this technology. In addition, AC is expensive and currently not approved for use as an animal feed additive; thus, alternative binding agents that bind androstenone and skatole are needed.

Skatole production has been confirmed in four bacterial species of two genera, *Clostridium* and *Olsenella*, that degrade tryptophan in the hindgut [8,9]. Skatole is then absorbed from the gastrointestinal tract and transported to the liver for subsequent metabolism. Skatole metabolites are then excreted in the urine or feces [10], while undegraded skatole is deposited partially in fatty tissue [11]. Reduction of skatole formation in the gut and accumulation in fat using dietary additives have been previously demonstrated. This included the use of fermentable carbohydrates such as inulin and raw potato starch, which increased the bacterial biomass and decreased the availability of tryptophan for skatole synthesis [9,12]. There is also an interaction between androstenone and skatole metabolism, where androstenone inhibits the expression of CYP2E1, a key enzyme involved in skatole metabolism [13]. Based on this interaction, decreasing the concentration of androstenone may also increase the metabolism and clearance of skatole.

Several inert adsorbents have previously been shown to reduce the effects of mycotoxicosis from contaminated feedstuffs in humans and livestock [14], including activated charcoal, various clays, zeolites, hydrated sodium–calcium aluminosilicates, and diatomaceous earth (as reviewed in [15]). Some of the most common mycotoxins are chemically similar to steroids such as estrogen and androstenone, in addition to the similarities in the biotransformation and enterohepatic circulation of such compounds [16]. These adsorbent materials bind mycotoxins to reduce their bioavailability in the gastrointestinal tract [17,18], further encouraging their potential application in binding boar taint compounds.

Bentonite (BNT) is a hydrated aluminum silicate of volcanic origin composed mainly of montmorillonite. It has both human and agricultural applications, including anticaking of feed pellets and as a filler for micronutrient premixes, as well as anti-inflammatory, dermatological, and cosmetic applications [19]. Diatomaceous earth (DE), also called diatomite or kieselguhr, is finely powdered silica of sedimentary origin and is composed of fossilized diatoms [20]. DE binds lipids and is used as an insecticide, a filtrate for a variety of liquids such as beer and syrup, an anticaking agent, and anti-parasitic treatment for companion and farm animals [21]. Spent filter aid (SFA) is a by-product from the use of DE in the corn syrup processing industry, with up to a third of its composition being DE and the rest made up of bioavailable nutrients, including crude protein and fat (~16–18% crude protein and ~34–42% crude fat, on a dry matter basis). This, along with its low cost (about two-thirds of the price of corn grain) as found locally, makes it an interesting multi-purpose coproduct in boar diets. Finally, hydrated sodium–calcium aluminosilicate (HCAS) belongs to a specific group of aluminosilicates or zeolites, which consist of various alkali metals and alkaline earth metals that determine their surface area, ionic charge, and ultimately, their capacities as adsorbents [15].

The purpose of this study was to evaluate several mineral adsorbents as alternatives to AC to bind skatole, androstenone (AND), estrone (E1) and estrone sulfate (E1S) in vitro. We assessed the binding capacity and affinity of four adsorbent additives (BNT, DE, SFA and HCAS) against AND, E1, E1S and skatole and analysed the data using multiple models (Michaelis–Menten, Langmuir and Freundlich isotherms). AC was also included as a positive control to support previous findings.

## 2. Materials and Methods

### 2.1. Preparation of Adsorbents and Adsorbates

Our methods were adapted from the study by Jen and Squires [22]. Stock solutions (40 mg/mL) of the adsorbents, bentonite (BNT; Sigma-Aldrich, St. Louis, MO, USA), diatomaceous earth (DE; Gaia Green Products Ltd., Grand Forks, BC, Canada), spent filter aid (SFA; Ingredion Canada, London, ON, Canada), hydrated sodium–calcium aluminosilicate (HSCAS, marketed as “Jumpstart 360^®^”; Jumpstart Animal Technology, Winnipeg, MB, Canada) and activated charcoal (AC; NORIT A^®^, Acros Organics, NJ, USA), were made in pH 7.4 phosphate-buffered saline (139 mM NaCl, 8.5 mM Na_2_HPO_4_, 1.5 mM KH_2_PO_4_, 2.7 mM KCl). Stock solutions (10 µg/mL) of the adsorbates androstenone (AND), estrone (E1), and estrone-1-sulfate (E1S) (Steraloids Inc., Newport, RI, USA) were prepared in PBS with 5% ethanol containing radiolabelled steroid ([3H]-5α-Androst-16-en-3-one (Moravek Biochemicals, Brea, CA, USA); [2,4,6,7-3H(N)]-estrone (Perkin-Elmer, Boston, MA, USA; 94.0 Ci/mmol), or [6,7-3H(N)]-estrone-1-sulfate (Perkin-Elmer, Boston, MA, USA)) at 15,000–18,000 cpm/mL. The androstenone stock solution also contained 0.05% Tween-60 surfactant (Sigma-Aldrich, St. Louis, MO, USA). The concentrations of the radiolabelled hormone solutions as determined above were well above the levels commonly found in intact males just before the onset of puberty. A stock solution of skatole (3 µg/mL) was prepared in PBS with 3% ethanol. This concentration was chosen based on studies involving weanling pigs, which found that pigs with cecal skatole concentrations above 3 µg/g in the feces resulted in the accumulation of skatole in adipose tissue above the boar taint threshold levels [23].

### 2.2. Dose–Response Curves and Adsorption Assays

The incubation conditions used were derived empirically by Jen and Squires [22] to determine the time taken to reach adsorption equilibrium between each adsorbent–adsorbate complex. Stock solutions of adsorbents were serially diluted with PBS, starting from 40 mg/mL to 20 mg/mL, 10 mg/mL, 5 mg/mL, 2.5 mg/mL, 1.25 mg/mL, 0.625 mg/mL, 0.312 mg/mL, 0.156 mg/mL, 0.078 mg/mL, and 0.039 mg/mL. Duplicate aliquots of 1 mL of each concentration were mixed with an equal amount of stock radiolabelled steroid absorbate solution (prepared as described above), capped, vortexed for 5–10 s, and then incubated for 30 min in a shaking water bath at 37 °C, with vortexing at the 15 min and 30 min incubation times. They were then centrifuged and 1 mL of the supernatant was mixed with 4 mL of Eco-Lite scintillator cocktail (ICN Biochemicals Canada Ltd., Mississauga, ON, Canada) and counted for radioactivity in a liquid scintillation counter (Beckman Coulter LS6000SC, Mississauga, ON, Canada).

To determine the binding of skatole, stock solutions of adsorbents were diluted in PBS to 40 mg/mL, 30 mg/mL, 20 mg/mL, 10 mg/mL, 5 mg/mL, 2.5 mg/mL, 1 mg/mL, 0.5 mg/mL, and 0.25 mg/mL. Duplicate aliquots of 0.5 mL of each concentration of adsorbent were mixed with an equal amount of 3 µg/mL stock skatole solution, capped, vortexed for 5–10 s, and then incubated for 10 h in a shaking water bath at 37 °C, with vortexing every hour. They were then centrifuged and a 200 µL aliquot of supernatant from each sample was diluted with 600 µL of PBS and the skatole levels were analysed by HPLC [21] using a C18 reverse phase column (Luna, 3 µnm 100 × 4.6 mm, ODS-2) and fluorescence detection with excitation at 285 nm and emission at 350 nm. The solvent system was 90% of 5 mM potassium dihydrogen phosphate (pH 3.9) and 10% *v*/*v* acetonitrile (buffer A), and 100% acetonitrile (buffer B), with a flow rate of 0.6 mL/min. The gradient consisted of the following: 0 min—70% A, 6 min—100% B, 11 min—100% B, 11.1 min—70% A and 17 min—70% A.

### 2.3. Michaelis–Menten Data Analyses

The binding of the adsorbent to the adsorbate was calculated from the difference between the amount of adsorbate in the supernatant after mixing with adsorbent and the amount of absorbate in a control solution with no adsorbent, expressed as a percentage of the total amount of adsorbate. To analyse the differences in binding, binding curves were generated and assessed for Michaelis–Menten parameters using the Enzyme Kinetics module of SigmaPlot 12.0, as described by Jen and Squires [22]. A modified Michaelis–Menten equation was used (Equation (1)):

(1) $$B=\frac{\left(B_{max})(C\right)}{K+C}$$

where B represents the percentage of adsorbate bound to adsorbent; B_max_ is the theoretical maximum of B (“binding capacity”); C is the concentration of adsorbent in mg/mL; and K is the affinity constant, i.e., the concentration of adsorbent at 50% of the B_max_ (analogous to the Michaelis constant Km in the original Michaelis–Menten equation). The experimental unit is the individual binding curve. All the statistical analyses were performed using ANOVA in SAS 9.4 (SAS Institute, Cary, NC, USA). The kinetic parameters were compared across adsorbents and adsorbates using Tukey’s range test.

### 2.4. Langmuir and Freundlich Isotherm Adsorption Model Analyses

Adsorption isotherm models produce a curve and parameters associated with the retention or mobility of an adsorbent to a solid phase in an aqueous medium at constant temperature and pH [24]. These are calculated at the adsorption equilibrium, when the adsorbent–adsorbate complex reaches an equilibrium between the amount adsorbed and the amount remaining in solution. The Langmuir and Freundlich isotherms are two of the most commonly analysed models used to describe and compare the affinity of adsorbents in solution. The choice of the appropriate model to best describe the binding behaviour can be performed through the goodness-of-fit parameters, such as the residual sum of squares (RSS) and/or the Akaike information criterion (AIC).

The Langmuir isotherm assumes that the adsorbed layer is one molecule thick and there are limited adsorption sites that are identical, with no adjacent interaction and steric hindrance between the molecules that are adsorbed (“homogeneous adsorption”), and it is described by Equation (2):

(2) $$\mathrm{Ca}=\frac{K_{2}K_{1}Cr}{1+(K_{1}Cr)}$$

where Ca is the amount of material adsorbed per unit mass of adsorbent (%); K_2_ is the maximum capacity of the sorbent material (%), and is equivalent to the B_max_ in Equation (1); K_1_ is the affinity of the adsorbent for each adsorbate studied (in mL/mg), also known as the dissociation constant, and is the inverse of K in Equation (1); and Cr is the concentration of adsorbent at equilibrium in mg/mL. The Langmuir model is very similar to the Michaelis–Menten equation as it predicts a constant monolayer capacity at high adsorbent concentrations [25].

The Freundlich isotherm is used to describe the reversible adsorption between the adsorbent and the adsorbate, and it applies multi-layer adsorption with a non-uniform distribution of the affinity with the adsorbate on the surface (heterogeneous adsorption). The model assumes that adsorption is the sum of all the occupied sites, with the binding sites for stronger affinities being occupied first until the energy of adsorption is exponentially decreased at equilibrium [24,26]. Equation (3) describes this:

Ca = K_F_C_r_^a^ (3)

where Ca is the amount of material adsorbed per unit mass of adsorbent (%); K_F_ is the isotherm constant (% (mg/mL)^a^) related to the capacity of the adsorbent for the material adsorbed; and a is the adsorption intensity, which describes the affinity of the adsorbent for the material adsorbed. Higher K_F_ and a values are desirable as they, respectively, reflect the greater capacity and affinity of the adsorbent.

Analyses of both the Langmuir and Freundlich models were conducted for each adsorbent across all the tested adsorbates using Microsoft Excel with the Solver feature developed by Bolster and Hornberger [27]. The residual sum of squares (RSS) and the Akaike information criterion (AIC) values were used to test for the goodness of fit and model appropriateness as produced by each non-linear model, and they are equivalent to R^2^ in the linear regression.

## 3. Results

**Figure 1:**
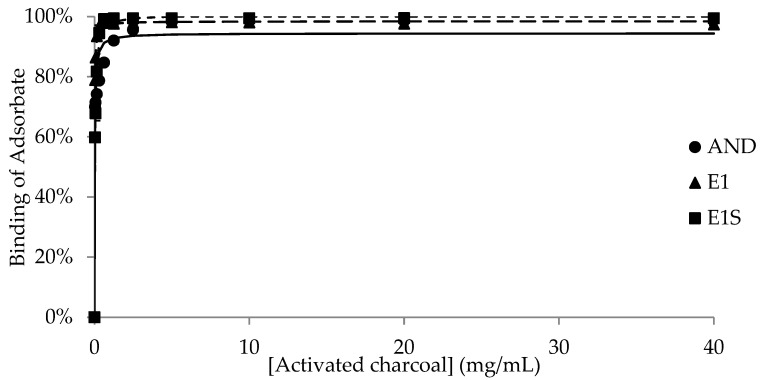
Binding of androstenone (AND), estrone (E1), and estrone sulfate (E1S) by 0.039–40 mg/mL of activated charcoal diluted in pH 7.4 phosphate-buffered saline.

**Figure 2:**
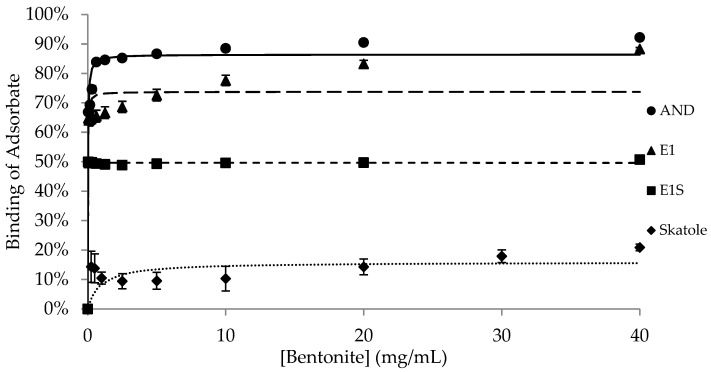
Binding of androstenone (AND), estrone (E1), estrone sulfate (E1S), and skatole by 0.039–40 mg/mL of bentonite diluted in pH 7.4 phosphate-buffered saline.

**Figure 3:**
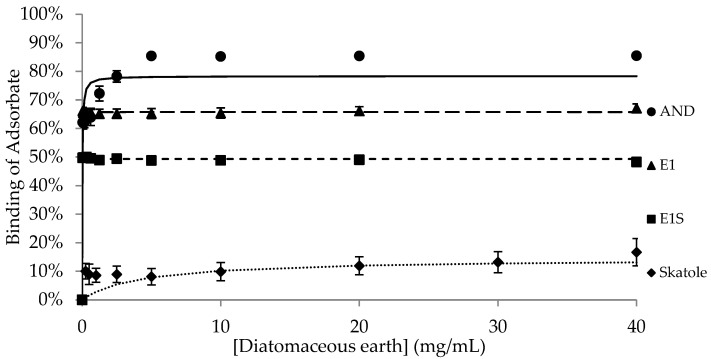
Binding of androstenone (AND), estrone (E1), estrone sulfate (E1S), and skatole by 0.039–40 mg/mL of diatomaceous earth diluted in pH 7.4 phosphate-buffered saline.

**Figure 4:**
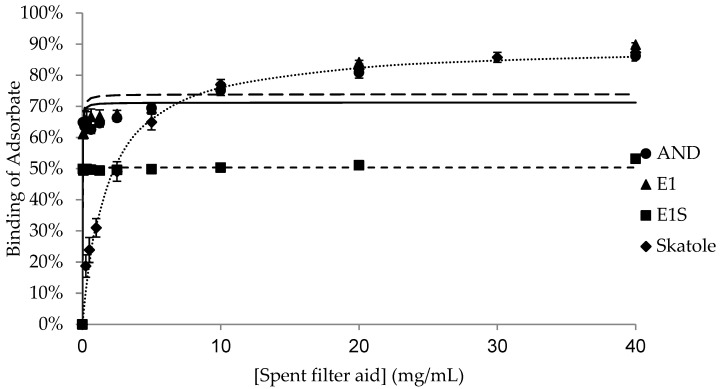
Binding of androstenone (AND), estrone (E1), estrone sulfate (E1S), and skatole by 0.039–40 mg/mL of spent filter aid diluted in pH 7.4 phosphate-buffered saline.

**Figure 5:**
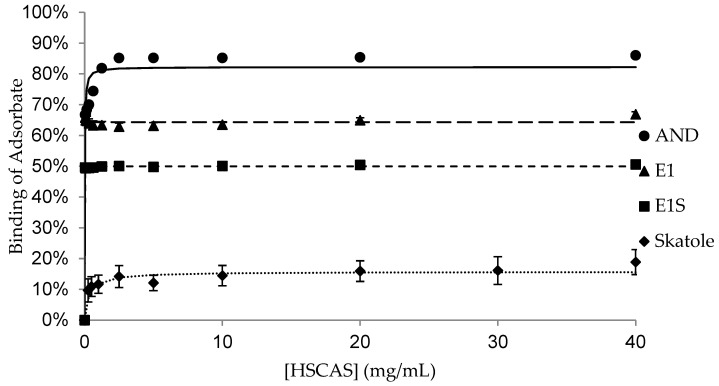
Binding of androstenone (AND), estrone (E1), estrone sulfate (E1S), and skatole by 0.039–40 mg/mL of hydrated sodium–calcium aluminosilicate diluted in pH 7.4 phosphate-buffered saline.

The binding of the adsorbates (AND, E1, E1S and skatole) to different concentrations of each adsorbent is shown in Figure 1, Figure 2, Figure 3, Figure 4 and Figure 5, with the detailed kinetic parameters describing the binding capacity (B_max_) and affinity (K) summarized in Table 1. The binding curves produced using AC as an adsorbent (Figure 1) support the results from previous experiments [21].

AC bound nearly 100% of E1 and E1S at 0.5 mg/mL, with respective B_max_ values of 97.3% and 103.7%, while AND was almost completely bound by AC at approximately 5 mg/mL, with a calculated B_max_ of 95.2%. AC completely bound skatole even at the lowest tested concentrations. AC had the highest maximum capacity of binding compared to all the other adsorbents (*p* < 0.05) (Table 1).

The affinity constant (K) for E1 binding by AC was significantly lower than the K value for AND and E1S (*p* < 0.05). The K values for any adsorbate did not differ between the adsorbents other than AC.

BNT and DE (Figure 2 and Figure 3) had significantly higher B_max_ values (77.7% and 71.9%, respectively) than SFA to bind AND (*p* < 0.05). SFA (Figure 4) had the lowest capacity for binding AND across the adsorbents, with a B_max_ of 55.0%, while HSCAS (Figure 5) was intermediate at 69.5% (Table 1).

Amongst the non-AC adsorbents, BNT and SFA had the highest maximum capacity for E1, with similar B_max_ values of 62.9% and 62.5%, respectively (*p* > 0.05). There was negligible and similar capacity for binding E1 by DE and HSCAS, with values of 2.6% and 0.3%, respectively (*p* > 0.05). Aside from AC, all the adsorbents bound E1S with very low capacity, between 0.4% and 4.3%, with no significant differences in binding across the adsorbents.

The binding capacity of SFA for skatole (Figure 4, 89.9%) was significantly higher than the B_max_ for skatole binding by BNT, DE, and HSCAS; the B_max_ was approximately 15% binding and not different among this group of adsorbents (Table 1). Since AC completely bound skatole even at the lowest tested concentrations, modelling with the Michaelis–Menten kinetics was not applicable.

The results concerning the Langmuir and Freundlich model isotherm parameters are summarized in Table 2. Adsorbent–adsorbate complexes with negligible binding were not assessed. This included the binding of E1S by BNT, binding of E1 and E1S by DE, binding of E1S by SFA, and binding of E1 and E1S by HSCAS. The columns are divided into the K_1_ and K_2_ values for the Langmuir isotherm, as well as the a and K_F_ values for the Freundlich isotherm. Goodness-of-fit regression analyses and model selection using the RSS and AIC were also included for each model. Lower RSS and AIC values indicate the better fit of a particular model over the other. Lower values for the RSS and AIC were found using the Langmuir model for the binding of AND by BNT; however, there was lower variation using the Freundlich model for the binding of E1 and skatole by BNT. All the tested compounds that bound to DE, SFA, and HSCAS favoured the Freundlich isotherm with the exception of binding of skatole by SFA, in which there were lower values using the Langmuir model. All the complexes between AC and its adsorbates favoured the Langmuir isotherm.

## 4. Discussion

In this study, we evaluated the binding of AND, skatole, E1 and E1S to the mineral-based adsorbents BNT, DE, SFA and HCAS compared to AC using an in vitro system at physiological pH. The objective was to find effective alternatives to AC for binding the boar taint compounds AND and skatole, which were also more cost-effective and had potential to be approved for use in animal feeds. The efficacy of binding was determined from a combination of the capacity (B_max_) and affinity (K) and the affinity (K, K_1_, and a) of an adsorbent to an adsorbate of interest; these parameters were obtained from a modified form of the Michaelis–Menten kinetics, in addition to the Langmuir and Freundlich isotherm adsorption models. All four mineral-based adsorbents bound AND, with the B_max_ values ranging from 55 to 78%, which were significantly lower than the binding of AND by AC, with a B_max_ of 95%. The binding of skatole was highest for SFA at 90%, while the other mineral-based adsorbents had a binding maximum for skatole of approximately 15%. BNT and SFA bound E1 with a B_max_ of approximately 62%, while there was negligible binding of E1 by DE and HSCAS. There was also negligible binding of E1S by any of the mineral-based adsorbents, while AC bound 100% of E1S.

This suggests that these mineral-based absorbents may selectively bind AND but not E1S in the gastrointestinal tract. They may therefore have less effect on the enterohepatic circulation of estrogens but may prevent the theoretical reabsorption of AND from the intestine into the blood stream. Decreasing the concentration of AND may be more important than that of skatole, as there are existing nutritional approaches to successfully control the production of skatole in the hindgut [28]. In addition, decreasing the concentration of AND would improve the metabolism and clearance of skatole by removing the inhibitory effect of androstenone on the expression of CYP2E1, a key enzyme involved in skatole metabolism [13]. Increasing the activity of CYP2E1 would contribute to lowering the concentration of skatole in fat.

Interestingly, the binding characteristics of DE and SFA were remarkably different. DE bound AND with a higher B_max_ than SFA did, but SFA bound skatole with higher B_max_ than DE did. DE did not effectively bind the estrogens, E1 or E1S, while SFA bound E1 but not E1S. SFA is comprised of DE bound to a layer of protein and fat, which is obtained from the filtration of corn syrup. These differences in surface chemistry may account for the differences in the binding parameters between DE and SFA.

BNT belongs to the phyllosilicates (“sheet-configuration”) family of clay minerals, which possess adsorptive ability through ionic exchange on active binding sites found on the surface and interior of layered silicates [15,29]. The binding capacity of AND by BNT was similar to the binding of AND by DE and HSCAS, while the binding of E1 by BNT was similar to the binding of E1 by SFA. The B_max_ values for the binding of BNT with E1, E1S, and skatole in this study were comparable to the results of Jen and Squires [22].

HSCAS, like BNT, is classified under the phyllosilicate group of clay minerals and features calcium ions and protons, which are exchanged against naturally occurring sodium ions [14]. The product tested in this study (“Jumpstart 360” produced by Jumpstart Animal Technology Ltd., Winnipeg, MB, Canada) also contains up to 10% propionic acid and activated lignite charcoal, in addition to HSCAS, and is marketed as an antifungal additive and odour control agent. HSCAS and BNT bound AND and skatole to a similar extent, while BNT bound E1 to a much greater extent than HSCAS did. These differences in binding may be in part due to the additional ingredients included in the Jumpstart product.

Parameters from the Michaelis–Menten, Langmuir, and Freundlich models all help to quantify and compare among the adsorbents’ capacity and affinity to bind compounds of interest. Adsorption isotherm models such as the Langmuir and Freundlich equations provide a useful quantitative contrast of mechanisms between various adsorbent–adsorbate complexes and are used here as another method of comparison in an in vitro system. These two isotherms are the most common models used for environmental contaminant-adsorbing materials analysis and aid in determining the behaviour of adsorptive surfaces as well as the interactions between adsorbed compounds on the surface. Based on the model fit criteria used, most of the non-AC adsorbent complexes fit better with the Freundlich model, indicating they are more appropriately described as possessing heterogeneous surfaces and are capable of reversible adsorption through multiple layered surfaces. However, it is essential to acknowledge the limitations of each isotherm. The Langmuir model was originally developed to compare the affinity of gas–solid-phase adsorption of activated charcoal [30]. This model does not take into account the process of displacement of pre-adsorbed molecules found in an aqueous solution (such as water) and only considers the adsorption of a studied compound, which questions its assumption of homogeneous binding [31]. The Freundlich model assumes an infinite number of adsorption sites, which does not allow determination of the maximum amount of adsorbates bound [25,26]. In addition, despite using these models, no standardized method exists for comparing between in vitro studies examining the same isotherms, making comparisons of binding affinities difficult across studies. No consistent set of error functions are used in relevant papers, and performing goodness-of-fit tests is dependent on whether or not the model has been linearized [24,27].

These in vitro binding experiments serve as an effective method to screen for adsorbent materials that could be added to finisher diets to control boar taint, but their dose in feed and the duration of feeding of treatment diets can only be assessed and validated using an in vivo feeding study. It is also important to acknowledge that in vitro results do not always readily convert to the same results in an animal model; an example of this is the inefficacy of activated charcoal to mitigate the effects associated with fumonisin B1 toxicity in rodents despite positive in vitro studies [18,32]. Similarly, feeding diets supplemented with fat-coated Biochar did not decrease the levels of skatole in the feces or plasma of finishing boars [33]. Furthermore, the direct transfer of in vitro results to determine the dietary inclusion levels of additives is complicated by the complexity of the intestinal environment. The charge distribution, polarity, and shape of the adsorbate are all important factors for the ultimate affinity of a mineral adsorbent [15]. It is also important to acknowledge that minerals can vary widely in composition depending on their origin and therefore this inconsistency can give mixed results.

For the ideal control of boar taint, the specific sequestration of AND and/or skatole is needed while minimizing the binding of other steroids in the gastrointestinal tract in order to limit any potential detrimental effects on intact male growth performance and carcass leanness. The binding of both boar taint compounds but not E1S by SFA makes it an ingredient of high interest to be included in future feeding trials. In addition, depending on its amount in feed, SFA may contribute a significant amount of protein and lipid to the diet, potentially offering additional benefits.

## 5. Conclusions

In summary, the mineral-based adsorbents BNT, DE, SFA and HCAS bound AND with B_max_ values ranging from 55 to 78%, while SFA bound skatole to a much greater extent than the other mineral adsorbents. While AC bound both boar taint compounds and estrogens at nearly 100% B_max_, the mineral-based adsorbents showed more specificity for binding AND with less binding of estrogens, which may be advantageous for their use as feed additives to control boar taint without affecting male growth performance.

## Figures and Tables

**Table 1:** Modified Michaelis–Menten kinetic parameters for individual adsorbents to bind boar taint compounds and estrogens.

	Adsorbent				
Parameter	BNT	DE	SFA	HSCAS	AC
AND					
B_max_	77.7 ± 1.12 ^a,1^	71.9 ± 1.93 ^a,1^	55.0 ± 7.85 ^b,1^	69.5 ± 1.44 ^ab,1^	95.2 ± 1.39 ^c,1^
K	0.1 ± 0.03	0.8 ± 0.43	0.8 ± 0.49	0.1 ± 0.05	0.1 ± 0.003 ^1^
E1					
B_max_	62.9 ± 7.69 ^a,1^	2.6 ± 1.68 ^b,2^	62.5 ± 8.29 ^a,1^	0.3 ± 0.22 ^b,2^	97.3 ± 0.86 ^c,1^
K	2.3 ± 2.20	1.7 ± 1.68	1.1 ± 1.02	N/D ^§^	0.03 ± 0.003 ^2^
E1S					
B_max_	4.3 ± 1.33 ^a,2^	0.4 ± 0.25 ^a,2^	2.9 ± 2.59 ^a,2^	0.8 ± 0.46 ^a,2^	103.7 ± 0.35 ^b,2^
K	26.5 ± 13.73	0.02 ± 0.02	4.4 ± 4.35	0.2 ± 0.15	0.1 ± 0.02 ^1^
Skatole					
B_max_	15.9 ± 1.55 ^a,2^	14.5 ± 3.82 ^a,3^	89.9 ± 1.09 ^b,3^	15.7 ± 3.24 ^a,3^	N/D ^‡^
K	0.9 ± 0.83	4.2 ± 4.15	1.8 ± 0.41	0.3 ± 0.13	N/D ^‡^

Values are represented as the mean ± SEM for 4 independent replicates. AND = androstenone; E1 = estrone; E1S = estrone sulfate; B_max_ = calculated maximum % of binding; K = concentration of adsorbent (mg/mL) to bind 50% of B_max_. BNT = bentonite; DE = diatomaceous earth; SFA = spent filter aid; HSCAS = hydrated sodium–calcium aluminosilicate; AC = activated charcoal. ^§^ The calculated K value for the binding of E1 by HSCAS was not different from zero; thus, it was unable to be determined. ^‡^ AC completely bound skatole at all the tested concentrations; thus, modelling with the Michaelis–Menten kinetics and comparisons were not possible. ^a,b,c^ Values within a row with different superscripts differ significantly at *p* < 0.05. ^1,2,3^ Values within a column with different superscripts differ significantly at *p* < 0.05.

**Table 2:** Parameters for the Langmuir and Freundlich adsorption isotherm models, and goodness-of-fit RSS/AIC values produced for the adsorption of AND, E1, and E1S and skatole.

		LANGMUIR				FREUNDLICH			
Adsorbent	Adsorbate	K_1_ (mL/mg)	K_2_ (%)	RSS	AIC	a	K_F_ (%)	RSS	AIC
BNT	AND	9.10	77.27	0.04	−52.43	0.12	58.12	0.05	−49.86
	E1	7.62	52.84	0.18	−35.69	0.18	37.00	0.03	−57.43
	Skatole	140.01	13.53	0.01	−48.30	0.09	11.60	0.01	−50.95
DE	AND	1.95	71.52	0.09	−44.03	0.19	41.12	0.06	−47.33
	Skatole	10.28	11.46	0.01	−55.81	0.12	8.85	0.003	−62.02
SFA	AND	10.70	46.63	0.20	−34.76	0.18	33.71	0.04	−52.14
	E1	5.14	54.59	0.18	−35.78	0.20	35.18	0.03	−55.74
	Skatole	0.58	89.70	0.01	−51.03	0.26	36.52	0.04	−37.82
HSCAS	AND	9.26	69.11	0.05	−49.49	0.12	52.46	0.05	−50.67
	Skatole	4.66	15.67	0.002	−63.34	0.11	11.51	0.001	−73.10
AC	AND	7.89	95.17	0.06	−47.08	0.12	70.30	0.08	−44.76
	E1	41.27	97.17	0.004	−76.55	0.04	88.01	0.08	−45.21
	E1S	9.57	103.56	0.04	−51.91	0.11	78.16	0.37	−27.94

Adsorbents. BNT = bentonite; DE = diatomaceous earth; SFA = spent filter aid; HSCAS = hydrated sodium–calcium aluminosilicate; AC = activated charcoal. Adsorbates. AND = androstenone; E1 = estrone; E1S = estrone sulfate. Parameters. K_1_ = Langmuir isotherm/dissociation constant; K_2_ = maximum capacity of sorbent material to specific adsorbate; RSS = residual sum of squares; AIC = Akaike information criterion; a = adsorption intensity; K_F_ = Freundlich isotherm constant. The Langmuir and Freundlich adsorption isotherm models were not tested for adsorbates when the binding was negligible.

## Data Availability

The original contributions presented in this study are included in the article; further inquiries can be directed to the corresponding author.
